# Continuous blood purification for severe acute pancreatitis: A systematic review and meta-analysis

**DOI:** 10.1097/MD.0000000000014873

**Published:** 2019-03-22

**Authors:** Yong Hu, Wenjun Xiong, Chunyan Li, Yunfeng Cui

**Affiliations:** a Tianjin Medical University, No. 22, Qixiangtai Road, Heping District; b Department of Surgery, Tianjin Nankai Hospital, Nankai Clinical School of Medicine, Tianjin Medical University, 122 Sanwei Road Nankai District, Tianjin, China; c Department of Medical Genetics, School of Basic Medical Sciences, Wuhan University, Wuhan, China.

**Keywords:** continuous blood purification, meta-analysis, organ failure, severe acute pancreatitis

## Abstract

**Background:**

The incidence of acute pancreatitis (AP) is rising around the world, thus further increasing the burden on healthcare services. Approximately 20% of AP will develop severe acute pancreatitis (SAP) with persistent organ failure (>48 h), which is the leading cause of high mortality. To date, there is no specific drug in treating SAP, and the main treatment is still based on supportive care. However, some clinical control studies regarding the superiority of continuous blood purification (CBP) has been published recently. Therefore, we conducted a systematic review and meta-analysis to evaluate the efficacy of CBP in SAP treatment.

**Methods:**

Four databases (Medline, SinoMed, EMBASE, and Cochrane Library) were searched for eligible studies from 1980 to 2018 containing a total of 4 randomized controlled trials and 8 prospective studies.

**Results:**

After the analysis of data amenable to polling, significant advantages were found in favor of the CBP approach in terms of Acute Physiology and Chronic Health Evaluation II (APACHE II) score (WMD = −3.00,95%CI = −4.65 to −1.35), serum amylase (WMD = −237.14, 95% CI = −292.77 to 181.31), serum creatinine (WMD = −80.54,95%CI = 160.17 to −0.92), length of stay in the ICU (WMD = −7.15,95%CI = −9.88 to −4.43), and mortality (OR = 0.60, 95%CI = 0.38–0.94). No marked differences were found in terms of C-reactive protein (CRP), alamine aminotransferase (ALT) and length of hospital stay (LOS).

**Conclusion:**

Compared with conventional treatment, CBP remedy evidently improved clinical outcomes, including reduced incidence organ failure, decreased serum amylase, APACHE II score, length of stay in the ICU and lower mortality rate, leading us to conclude that it is a safer treatment option for SAP. Furthermore, relevant multicenter RCTs are required to prove these findings.

## 1 Introduction

Acute pancreatitis (AP), one of the most common gastrointestinal diseases worldwide,^[1,2]^ is an inflammatory disease initiated by intra-acinar activation of proteolysis caused by pancreatic enzymes. AP causes a source of substantial service burden and hospital cost in nearly all countries.^[3,4]^ Gallstones and alcohol abuse are the main prevalent causes of AP. In addition, hyperlipidemia, hyperkalemia, anatomic variation and idiopathic acute pancreatitis (IAP) act as other factors for AP.^[5,6]^ The 2012 revised Atlanta classification divides AP into 3 clinical severity levels of mild, moderate and severe.^[7]^ More than half of patients with AP will develop edematous pancreatitis with a mild course, which is a self-limiting disease that resolves with conservative medical management, requiring only a brief period of hospitalization.^[8]^ SAP accompanied by necrosis of the (peri) pancreatic tissue and (multiple) organ failure (MOF), with a mortality of at least 30%, is still a challenge in the medical field even with the ever-progressing level of medical treatment.^[9]^ Currently, there is no clear indicator of the development of severe pancreatitis.^[10,11]^ The clinical course of SAP can be divided into 2 phases. In the first phase, systemic inflammatory response syndrome (SIRS) and MOF occur frequently and are the main cause of death.^[12]^ With the gradual insight of its pathogenesis, SAP induces elevated levels of tumor necrosis factor-alpha (TNF-alpha) and Interleukin-1B (IL-1b) in the circulatory system and further induces production of IL-6 and IL-8. This, in turn, leads to hypercytokine, SIRS, shock, loss of internal dynamic balance, and organ dysfunction.^[13–15]^ Therefore, preventing and blocking the occurrence and progression of SIRS is the key to the treatment of SAP. There have been some clinical studies on blood purification therapy for sepsis and SAP, but there is not enough evidence to prove the superiority of this therapy. Hence our aim is to examine the clinical effects of CBP in the treatment of SAP in this systematic review and meta-analysis.

## 2 Methods

Ethical approval or patient consent was not required because the present study was a review of previously published articles.

### 2.1 Search strategy and study selection criteria

A computerized search spanning the years 1980 to 2018 was conducted in Medline, Sino Med, EMBASE, and Cochrane Library databases. The following search terms were used in all possible combinations: (“Pancreatitis ”[Mesh] OR “Pancreatitis, acute “[Mesh] OR ”Pancreatitis, multiple organ dysfunction syndrome“[Mesh]) AND (”blood purification“[Mesh] OR “high-volume hemofiltration ”[Mesh]) and “continuous veno-venous hemofiltration”. The search was limited to human subjects. There was no language limitation. The titles and abstracts of potentially relevant studies identified by the computerized search were reviewed. Full-text articles were obtained for detailed evaluation, and eligible studies were included in the systematic review. The findings of NRS may also be useful to inform the design of a subsequent randomized trial. The inclusion criteria were the following: both RCTs and observational clinical trials; the study included patients who were of either sex, had a clinical diagnosis of SAP; CBP should be administered as the treatment, with the aim of the trial being a comparison of the CBP and control group in treating SAP; the outcomes should be clearly described including at least 1 of 5 major outcomes, such as the levels of APACHE II, serum amylase, serum creatinine, LOS, and incidence of mortality.

The exclusion criteria were the following: absence of comparison between CBP and conventional treatment group; absence of the characteristics of patients and missing information data about treatment outcome data, which were insufficiently clear; clinical experience and case reports.

### 2.2 Data collection and extraction

Two authors independently extracted data from reviewing all titles and abstracts of the searched papers. The following information was recorded from the included trials: first author, year of publication, number of participants. Basic data about gender, age, etiology, APACHE II score, and diagnosis were extracted and analyzed. To compare the levels of APACHE II, serum amylase, serum creatinine, clinical outcomes of the mortality and LOS, we used a formula adopted by previous studies to acquire the mean and standard deviation (SD). According to these criteria, 2 independent reviewers reached a consensus whenever discrepancies arose and performed identification and selection of the studies. The selection process was documented according to PRISMA criteria.

### 2.3 Quality assessment and risk of bias

Two readers independently extracted and reviewed the data from the enrolled studies to ensure consistency. The quality of the included RCTs, as assessed by the Cochrane Handbook for Systematic Reviews of Interventions, and quality assessment of the included retrospective trials, assessed by the Newcastle–Ottawa Scale. Egger test was used to assess publication bias, which was based on the OR of mortality in severe acute pancreatitis.

### 2.4 Statistical methods

For alignment outcomes, the number of patients for each treatment outcome was used in the analysis. Odds ratios (ORs and variances) for the 8 different complications comparing CBD and conventional treatment were calculated for each comparative study. The associated log ORs were meta-analyzed using a restricted maximum-likelihood random effects model, after which the results were transformed back into the OR metric. Heterogeneity of ORs across studies was examined and considered where present, but the random-effects model was used regardless of whether there was significant random-effects variation. Fixed effects model was performed as a sensitivity test. The study included both randomized clinical trials and observational studies, and subgroup analyses were used to investigate heterogeneous results based on different study types. All statistical analyses were conducted using STATA 14.0. For dichotomous outcomes in the extracted data, OR and 95%CI were calculated, and WMD were used for continuous outcomes. When the interquartile range and median were given instead of the SD, we converted the data using the Hozo algorithm to estimate the SD.^[16]^

### 2.5 Sensitivity analyses

We performed sensitivity analysis to assess the stability of the results and investigate the influence of each study by omitting a single study sequentially. Publication bias was shown by funnel plot. Using the Egger test, we found no evidence of bias in any of the lag periods.

## 3 Results

### 3.1 Included trial characteristics and quality assessment

**Figure 1:**
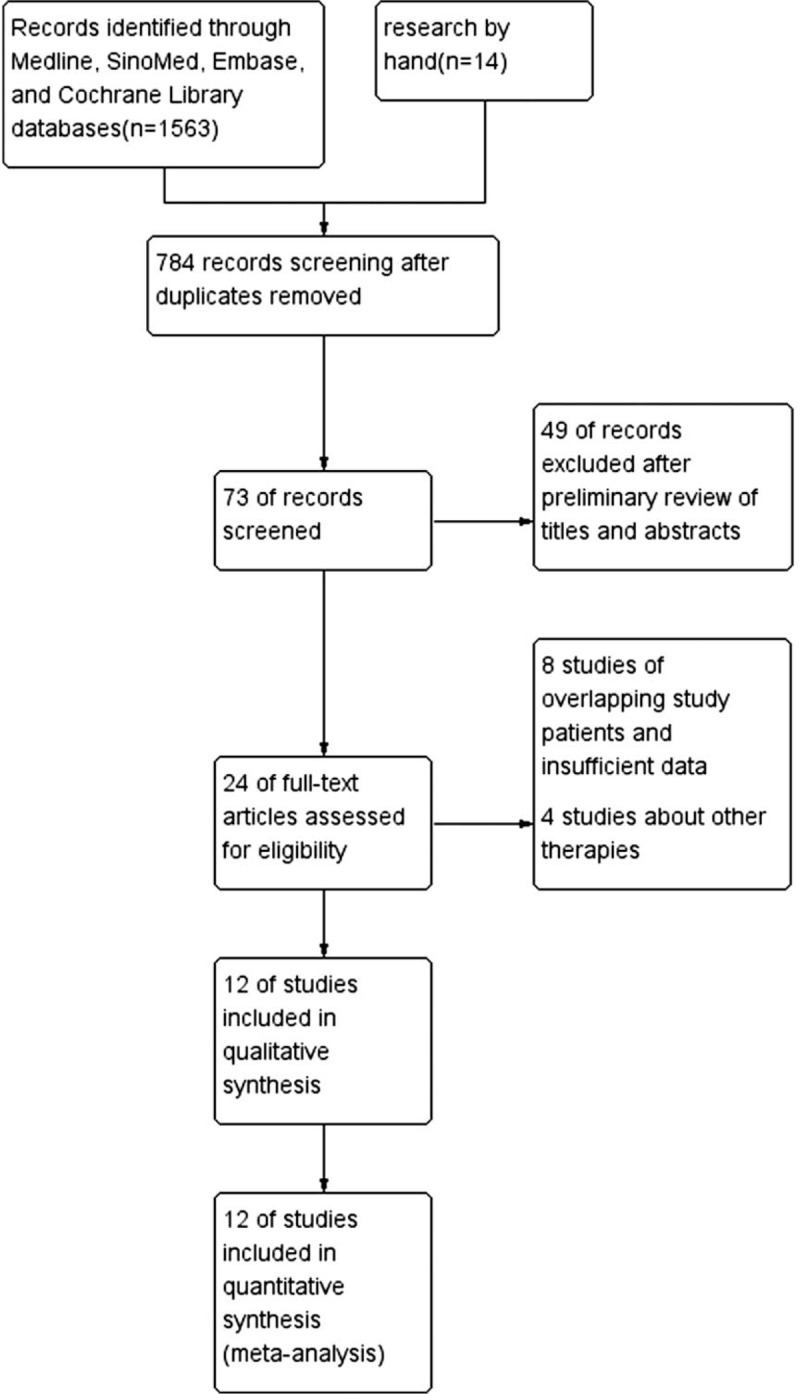
Flow diagram for selection of studies for inclusion in this meta-analysis.

The initial 1563 citations were identified based on a study of the subject and a summary of the literature, of which 784 articles were thereafter excluded because of duplication. After reviewing the title and abstract of the remaining 73 studies, only 24 full-text studies were evaluated for further assessment, and 11 obviously irrelevant records were excluded. Eventually, 12 clinical studies were consistent with the inclusion requirements.^[17–28]^ A detailed study flow-diagram is shown in Figure 1.

The characteristics of the included studies were illustrated in Table 1. The quality of the included RCTs was assessed by the 7-point Modified Jadad Score and the quality assessment of the included retrospective trials was assessed by the 9-star Newcastle-Ottawa Quality Assessment Scale.

**Table 1:**
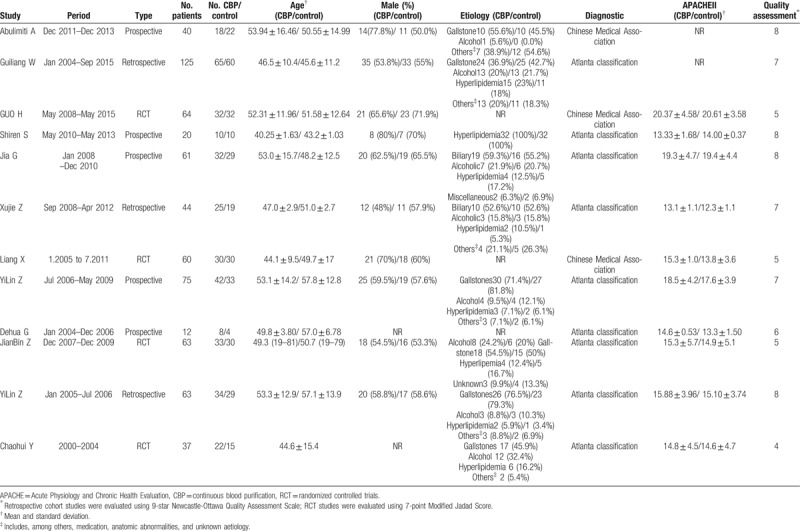
Main characteristics of the inclusion studies.

### 3.2 Details of the trial process

Twelve studies were selected, with a total of 664 patients (351 patients underwent CBD and 188 patients underwent conventional treatment) included. Two randomized control trials investigated the effect of continuous veno-venous hemofiltration (CVVH) in patients with severe acute pancreatitis. The other 2 RCTs evaluated HVHF for the treatment of severe acute pancreatitis. Five prospective cohort studies compared early HVHF with the conventional method in the treatment of SAP, which recorded the mortality, LOS and other complications. The other 3 retrospective studies reported on the different outcomes of HVHF, CVVH and conventional method in treating SAP. All studies detailed the pre-treatment biochemical and scoring values of patients and recorded the major outcomes (LOS, mortality).

### 3.3 Meta-analysis results

**Figure 2:**
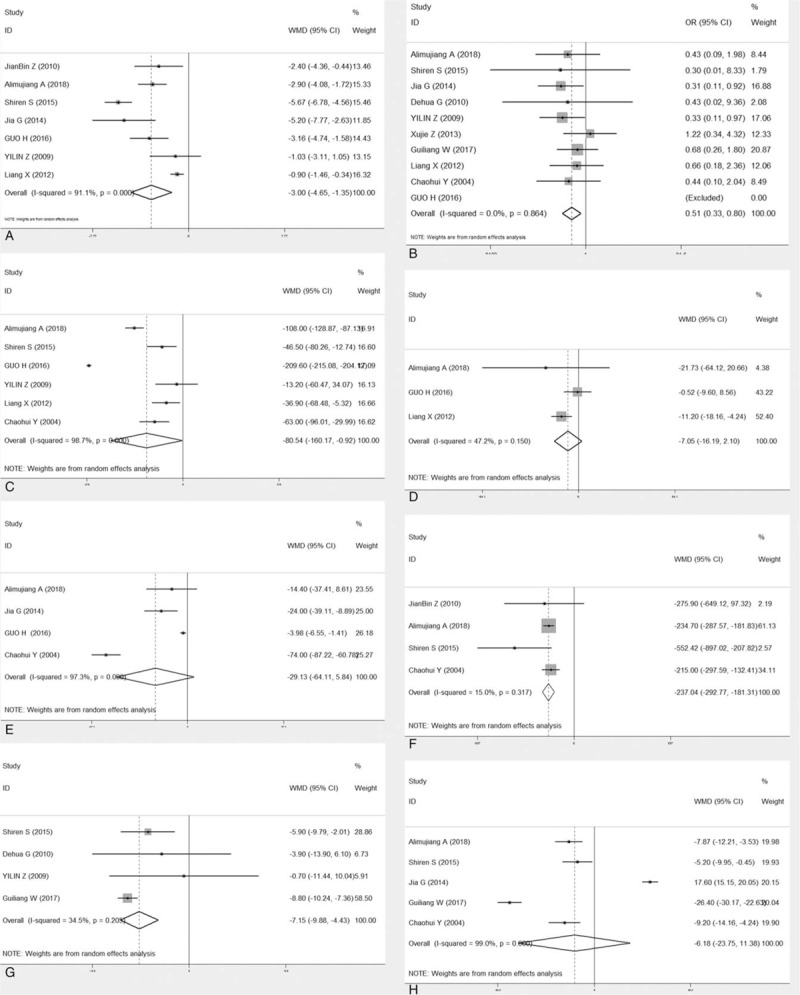
Forest plot (whole study) of the merits between continuous blood purification and the conventional treatment in the light of clinical outcomes. (a. APACHE II score. b. Mortality. c. Serum creatinine. d. Alamine aminotransferase. e. C-reactive protein. f. Serum amylase. g. Days in intensive care. h. Length of stay in hospital.) CI = confidence interval, OR = odds risk.

**Figure 3:**
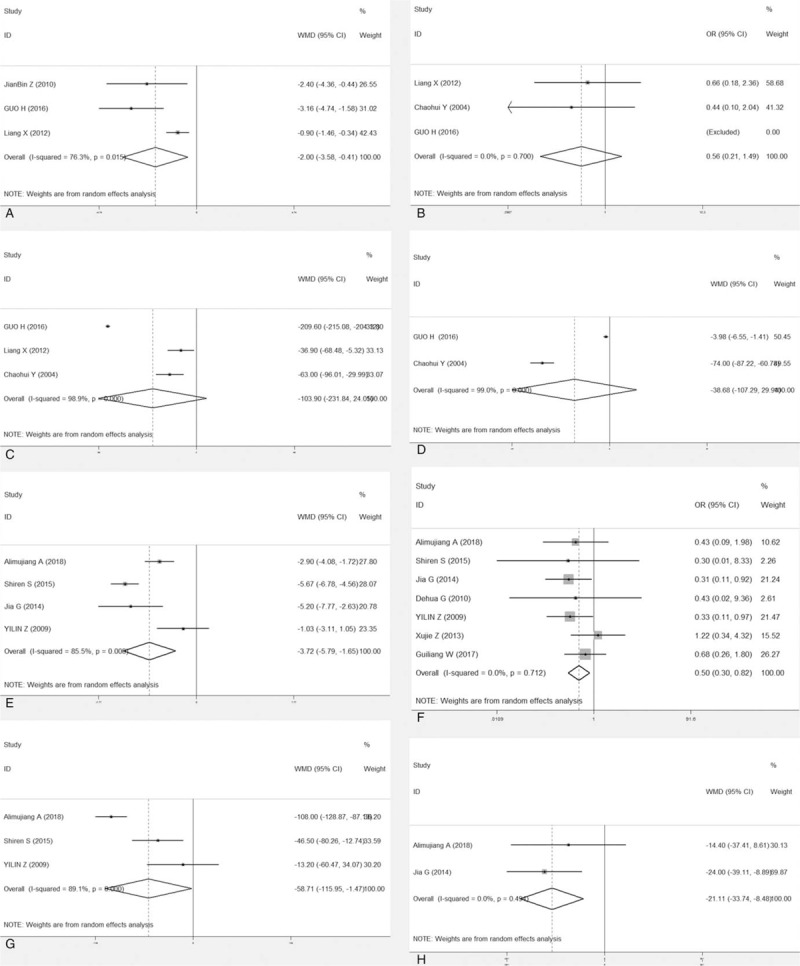
Forest plot (subgroup, divided by RCT and retrospective study) of the merits between continuous blood purification and the conventional treatment in the light of clinical outcomes. (a. RCT- APACHE II score. b. RCT-mortality. c. RCT-serum creatinine. d. RCT- CRP. e. retrospective study- APACHE II score. f. Retrospective study-mortality. g. Retrospective study-serum creatinine. h. Retrospective study- CRP). APACHE II = Acute Physiology and Chronic Health Evaluation II, CI = confidence interval, CRP = C-reactive protein OR = odds risk, RCT = randomized controlled trial.

Eight studies reported APACHE II score, 4 studies recorded serum amylase levels, CRP, ALT, and length of stay in the ICU, and 6 studies showed serum creatinine and LOS, and 10 studies reported rate of mortality. All studies provided incidence data of at least 1 kind of complication. CBD was superior to conventional treatment with respect to APACHE II score, serum creatinine, and serum amylase. CBD demonstrated significant lower incidence of mortality and length of stay in the ICU when compared to conventional treatment, with OR0.60 (95% CI, 0.38–0.94) and WMD −7.15 (95% CI, −9.88 to −4.43) for mortality and length of stay in the ICU, respectively. However, the days of hospital stay, ALT and CRP did not show significant advantages WMD −6.18 (95% CI, −23.75 to 11.38), −7.05 (95% CI, −16.19 to 2.09) and −29.13 (95% CI, −64.11 to 5.84) (Fig. 2). According to the stratified analysis of results from the RCT and retrospective studies (Fig. 3), there is no difference in mortality rate between CBP and conventional treatment based on the RCT studies only, with an OR of 0.56(95% CI, 0.21–1.49). However, based on the retrospective studies, CBP group has a significantly lower rate of mortality than conventional treatment group, with an OR of 0.50 (95% CI, 0.30–0.82). Based on stratified analysis results of the RCT and retrospective studies, we noted a significant difference between CBP and conventional treatment with respect to APACHE II score, length of stay in the ICU or mortality rate.

### 3.4 Sensitivity analysis and publication bias

Sensitivity analysis was performed to assess the stability of pooled results. Among the 12 studies, the significant results were not obviously altered after sequentially omitting each study. In the pooled results comparing the incidence of mortality, after excluding the report of Alimujiang et al, the heterogeneity decreased significantly (OR = 0.719, 95%CI = 0.277–1.865, *P* = .497, *I*^2^ = 28%) and showed that there was no significant difference in preventing the mortality rate between the 2 groups; hence, it was regarded as a result of heterogeneity. Likewise, the other studies were considered as the source of heterogeneity because the heterogeneity significantly changed and showed that there was no significant difference in preventing the mortality rate between the 2 groups by excluding each of these studies in the pooled results comparing the incidence of mortality. A sensitivity analysis was conducted to determine whether the exclusion of this study would alter the result, and exclusion of this study from the meta-analysis did not substantially influence the results.

**Figure 4:**
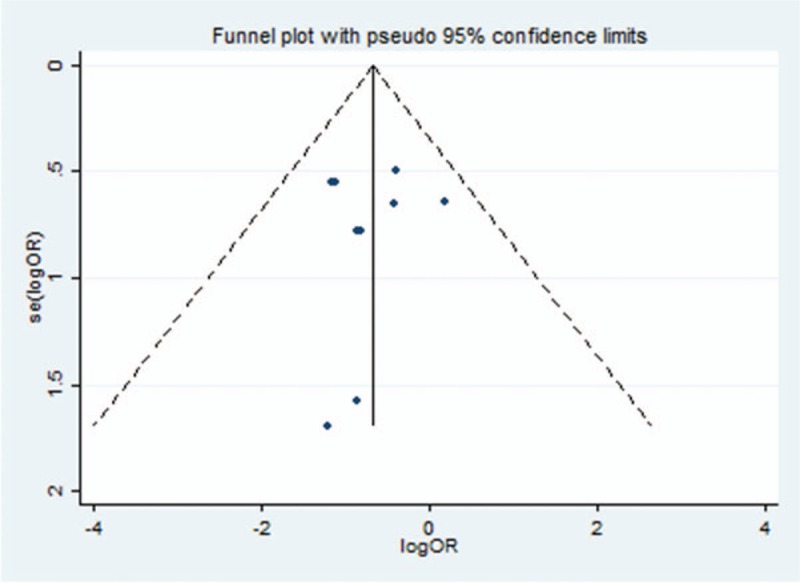
Funnel plot of 2 intervention for outcome of mortality. OR = odds risk, SE = standard error.

In this part of the study, 4 RCTs and 8 prospective trials were included. The funnel plots of the ORs for mortality and necrotizing pancreatitis were used to assess publication bias. Egger test results showed Pr > jzj = 1.00 (Fig. 4). Therefore, we believe that the risk of publication bias is low in this meta-analysis.

## 4 Discussion

### 4.1 Summary of the main results

Severe acute pancreatitis accounts for 20% of AP cases and is associated with a high mortality and morbidity.^[29,30]^ With the rising incidence of SAP, feasible and effective management is greatly needed. At present, supportive care (e.g., fluid resuscitation, tracheal intubation) plays a pivotal role in the treatment of SAP.^[31]^ Which type of treatment represents the most effective is discussed controversially. However, many studies have been published on the preponderance of CBD, including the CVVH and HVHF approach.^[32–35]^ But these documents have their own shortcomings, such as the small number of patients and missing multi-center research. Therefore, the focus of this analysis was to evaluate the efficacy of CBP approaches in the treatment of SAP. The meta-analysis identified 12 published studies that assessed the outcomes of patients with SAP who underwent CBP or conventional treatment. There are few published RCTs because of the lack of patients, necessary equipment, and technically savvy experts in addition to the presence of uncontrollable risk during treatment. Much evidence of effects cannot be adequately studied in randomized trials, such as long-term and rare outcomes. Therefore, we analyzed all cohort studies in this study. For the main results, there was no notable difference in CRP and LOS between the 2 methods in patients with SAP. Improved clinical outcomes, including reduced incidence of mortality and decreased length of stay in the ICU, were detected in patients who underwent a CBP approach.

### 4.2 Comparison with previous studies

In consideration of the long history of medical development and AP's widespread occurrence, the best treatment has obviously improved. Many large-scale multicenter studies have found that early active fluid resuscitation, early enteral nutrition, rational use of antibiotics and minimally invasive surgical treatment can effectively reduce mortality in severe acute pancreatitis.^[35–39]^ but there is a lack of large sample studies on the treatment of severe acute pancreatitis with blood purification. Similarly, there is no meta-analysis published on blood purification for severe acute pancreatitis. Therefore, this is a novel systematic review and meta-analysis. Due to the inadequate evidence, we present this meta-analysis by consolidating multiple studies to enable enhanced clinical decision making in the future.

### 4.3 Limitations of the study

However, despite a comprehensive analysis, there are also many limitations that should be taken into consideration in our meta-analysis. First, the studies included in the meta-analysis were not all RCTs. Second, in the literature-included studies, every study in the CBP approach is not completely similar. Third, all studies were from the Affiliated Hospital of Chinese universities. Fourth, partial missing information in a few articles may lead to biased results. We have attempted to contact investigators or study sponsors to verify key study characteristics and obtain missing numerical outcome data. In addition to the portions of the studies that did not directly provide means and SD, the authors used Hozo algorithm to estimate those values; this may have introduced bias. Moreover, clinical and method logical heterogeneities were observed in several parameters in the meta-analysis given the variation in intervention techniques, patient composition, and preferences among different centers. True heterogeneity and poor methodological quality could also lead to an asymmetric plot. In the future, larger, higher quality clinical trials comparing the 2 approaches should be conducted, and we will conduct a more detailed subgroup analysis to explore the sources of heterogeneity to obtain a more reliable conclusion.

## 5 Conclusion

In summary, we demonstrated that improvement in several clinical outcomes, including APACHE II, serum amylase, serum Cr, length of stay in the ICU and mortality were recorded in SAP patients who underwent CBP treatment. Thus, we conclude that the CBP approach is a safe and effective treatment option for patients suffering from SAP. There is a great need for more RCTs to confirm these advantages. In addition, future studies will be required to further define the optimal time interval and techniques for the continuous blood purification procedure.

## Author contributions

**Data curation:** Yong Hu, Wenjun Xiong.

**Formal analysis:** Yong Hu, Chunyan Li.

**Writing – original draft:** Yong Hu.

**Writing – review & editing:** Yunfeng Cui.
